# Community Mobilization and the Framing of Alcohol-Related Problems

**DOI:** 10.3390/ijerph7031226

**Published:** 2010-03-22

**Authors:** Denise Herd

**Affiliations:** School of Public Health, 50 University Hall, University of California, Berkeley, CA 94720, USA; E-Mail: tiara@berkeley.edu; Tel.: +1+510 642 4842; Fax: +1-510-643-8236

**Keywords:** alcohol policy, social movements, collective action frames, alcohol outlets, urban populations

## Abstract

The goal of this study was to describe how activists engaged in campaigns to change alcohol policies in inner city areas framed alcohol problems, and whether or not their frameworks reflected major models used in the field, such as the alcoholism as a disease model, an alcohol problems perspective, or a public health approach to alcohol problems. The findings showed that activists’ models shared some aspects with dominant approaches which tend to focus on individuals and to a lesser extent on regulating alcohol marketing and sales. However, activists’ models differed in significant ways by focusing on community level problems with alcohol; on problems with social norms regarding alcohol use; and on the relationship of alcohol use to illicit drugs.

## 1. Introduction

Over the past several decades, various grassroots organizations in the United States have mobilized to challenge alcohol policies in inner city neighborhoods. These groups have developed local and statewide ordinances to limit and regulate alcohol outlets, organized networks to eliminate alcohol billboard advertising, and launched protests against racial and ethnic targeting by alcohol and tobacco companies [1–4]. However, few studies have focused on this social movement or have analyzed the ways in which it has defined, or framed, alcohol issues to mobilize constituents. As a result, little is known about how activists in this movement conceptualize alcohol problems and whether the constructs they use coincide with or differ from some of the major frameworks used in contemporary social policy discussions.

The literature on social movements suggests that understanding collective action frames, or the “action-oriented sets of beliefs and meanings that inspire and legitimate the activities and campaigns of a social movement organization” [5] (p. 614), is critical for analyzing how these kinds of movements develop, and ultimately for understanding why they succeed or fail [6,7]. The importance of framing in public-health-related social movements has been exemplified in movements related to drinking and driving, tobacco control, and homelessness. For example, studies have described how framing and problem construction facilitated the sweeping success of the 1980s anti-drunk driving movement, despite few changes in the actual rates of drinking and driving or resulting injuries, accidents, and deaths [8,9]. Similarly, Derry and Waikar [10] demonstrated that antismoking mobilization can be understood in terms of the contrasting frames used by the tobacco industry and public health activists (*i.e.*, the former uses a master frame to portray its honesty, with supporting core frames citing the uncertainty of health risks; and the latter use a master frame of distrust for the industry and core frames citing the substantial health risks of smoking) [10]. Cress and Snow [11] studied mobilization among organizations that serve the homeless and concluded that framing processes were necessary to achieve successful social movement outcomes.

### 1.1. Key Approaches to Framing Alcohol-Related Problems

A number of frameworks have been widely used by alcohol policy researchers [8,9,12] to define the nature of and causes of alcohol-related problems. These frameworks include the *alcoholism as an addictive disease* model, the *alcohol-related problems* framework, the *personal responsibility and blame* model, and the *public health* framework for addressing alcohol problems.

The alcoholism as an addictive disease paradigm became the dominant model for conceptualizing alcohol-related problems in the US after World War II. This approach reflects strong anti-prohibitionist sentiment and focuses primarily on the problems of addiction within the individual. In this model, alcoholism is regarded as a loss of control over alcohol in biologically predisposed individuals who experience a myriad of health and social problems as a result of their addiction. Individual alcoholics are believed to be the main source of society’s problems with alcohol, and providing adequate alcoholism treatment is viewed as the main public policy solution for handling alcohol-related problems [12].

Although the alcoholism as a disease model was immensely influential and still shapes some clinical and lay people’s understanding of alcohol problems, by the 1970s, the scientific community began to question the validity of bundling such a wide range of problems under the rubric of alcoholism. As Room [13] noted, a 1979 report to Congress stated that “alcohol problems in the general population do not seem to form a coherent pattern. The problems are too diffuse to be described as part of a single concept of alcohol addiction” (p. 62). A National Academy of Sciences report echoed similar themes, pointing out that although heavy drinkers exhibit the highest rates of alcohol problems, a larger number of low-quantity drinkers in absolute numbers account for more alcohol-related problems [2]. Compared with the disease paradigm, this disaggregated approach to alcohol problems requires a broader spectrum of strategies (e.g., preventing drinking and driving and other types of injuries) to address the myriad health and social problems related to alcohol.

The personal responsibility and blame model for alcohol problems was popularized during the wave of citizen activism regarding drinking and driving in the early 1980s. This model targets the individual drinker as deviant and criminal for violating laws and harming others. As Renairman [8] stated, “MADD’s organizing strategy assiduously avoids attention to corporate and structural sources of alcohol problems in favor of a rhetoric of individual responsibility, the private moral choice of drinkers, and solutions based upon both self-regulation by both drinkers and alcohol, advertising and broadcast industries” (p. 105).

In contrast, traditional public health models [8,13,14] prioritize the roles of alcohol beverage availability, distribution, sales and marketing, and consumption as the key factors in determining levels of alcohol-related problems within the society. The key levers for reducing or preventing alcohol-related problems in these models are regulating the sales and distribution of alcohol (e.g., through price controls, restrictions on sales venues and hours) and limiting the demand for it (e.g., through curtailing advertising). From this perspective, the alcohol industry and government policies regarding alcohol availability, rather than the individual drinker, are regarded as the major loci of responsibility for society’s alcohol problems.

### 1.2. Research Questions

The present study explored how activists defined alcohol problems and what they viewed as the most important alcohol-related problems in their communities. Our focus on problem definition reflects the importance of diagnostic framing, as described by Cress and Snow [11]. Diagnostic framing focuses on articulating the genesis of a problem and on identifying who or what is to blame; as such, it contrasts with prognostic framing, which focuses on articulating solutions to that problem.

This was an exploratory study, and the major goal of the analyses was to provide a descriptive account of the key conceptual frameworks used by those who led local or regional alcohol policy campaigns in seven urban areas across the US. Three central research questions guided the analyses.

First, how did activists define and interpret alcohol problems? Given that the campaigns generally focused on regulating the sales, marketing, and advertising of alcoholic beverages, we expected that many activists would describe public health definitions of alcohol problems, rather than approaches emphasizing alcohol addiction or abuse, or the problems of individual drinkers.

Second, what was the perceived importance for activists of different kinds of problems related to alcohol use? Our goal was to ascertain whether some issues had more salience than others with respect to how activists framed alcohol problems. Again, given their policy goals, we expected that activists would rank public-health-oriented alcohol-related problems more highly than problems at the clinical or individual level.

Third, were there significant differences in how alcohol problems were defined or ranked, based on the roles of the activists or on differences in the communities in which they worked? The respondents were from diverse backgrounds, and their respective communities confronted different kinds of problems, which could have lead to differences in how problems were framed. For example, the respondents included personnel who worked in alcohol treatment agencies or were in recovery, as well as law enforcement officers. Both of these groups might be expected to favor models other than the public health approach (e.g., the alcoholism as a disease framework or the personal responsibility and blame model). In addition, the communities addressed different kinds of problems (e.g., excessive rates of drinking under the influence [DUI], alcohol addiction, and homelessness) that might have predisposed activists working in different sites to espouse different frameworks.

## 2. Experimental Section

Data for this study were based on the responses of activists who were interviewed in neighborhoods in seven US cities, including Oakland and Los Angeles, California; Milwaukee, Wisconsin; San Antonio, Texas; Raleigh, North Carolina; Detroit, Michigan; and Baltimore, Maryland. These cities were chosen because they all had least a 5-year history of activism regarding alcohol policy issues, were located in different parts of the US, and included activists working on a range of alcohol policy issues. The cities were selected on the basis of interviews with several key informants who had worked extensively on community-based alcohol policy issues in the US, as well as through examining newspaper records of activism regarding alcohol policy in particular sites.

All of the communities selected were actively engaged in efforts to change local ordinances and/or statewide laws regarding the sale or marketing of alcoholic beverages. They focused on issues such as limiting the amount of billboard advertising devoted to alcoholic beverages and reducing or exercising greater control over liquor stores or licenses. All of these communities achieved at least some of their goals. Collectively, at least six laws were created or changed at the state level; in Los Angeles, 270 alcohol outlets surrendered their licenses after the civil unrest, and due to community activism many of them did not re-open [15]; and billboards advertising alcoholic beverages or tobacco products were taken down in other cities. These goals were achieved through a variety of strategies, including public awareness and educational campaigns, and the forging of relationships between activists and the media, elected officials, and a broad range of community organizations. In many cases, framing of alcohol problems was central to the development of effective strategies. For example, activists from three of the communities (Milwaukee, Oakland, and San Antonio) pointed out that, at the beginning of their movement, they often were mistakenly labeled as prohibitionists; in the words of one activist, they were seen as individuals who were “trying to take away my six-pack of beer after work,” rather than as individuals trying to offer an alternative to the destructive force alcohol can have on a community. Reframing was an important aspect of public awareness, and played a large role in successfully mobilizing communities around efforts for change.

Informants from each site were selected using snowball sampling techniques described by Luker [16] in her study of pro-life and pro-choice activists. Potential participants for each area were identified primarily by consulting with community organizers and advocates who had worked with community groups on alcohol policy issues and were familiar with key activists, and by examining newspaper coverage of alcohol policy activities that mentioned community leaders. To be included in the study, each potential informant had to be recommended by at least two people as an individual who could be considered an important leader regarding alcohol policy work in his or her community. When neighborhood leaders were contacted or interviewed, they were asked if they knew of other people who played an important role in local campaigns regarding alcohol whom we could contact. We continued the process of asking for referrals and creating lists of people recommended by a least two sources until we reached the point at which no new names were being submitted. We invited these individuals to participate in the study and followed up by informing them about the study and scheduling interviews. Through working with local leaders who supported the goals of the study, we obtained permission from and completed interviews with most (70% or more) of those invited to participate in the study. Most of the interviewees were still actively working on alcohol policy issues at the time they were interviewed.

A total of 184 activists were interviewed across the seven sites. The interviews and fieldwork took place from August 1996 through the end of 1999. About 40 activists were interviewed in both Oakland and Los Angeles, 28 in Milwaukee, and 17 to 21 activists in Raleigh, San Antonio, Detroit, and Baltimore. A little more than a third of the activists were classified as community or neighborhood activists. Neighborhood activists usually volunteered their time, in contrast with those described as professionals (41%), who worked with alcohol services for pay in the areas affected by alcohol use and policy, such as law enforcement, education, city planning, and law. Ten percent of the interviewees were local or state politicians, six percent were clergy, and seven percent were classified in other categories. The majority of leaders interviewed were African American (67%), although whites (16%) and Latinos (14%) also were significantly represented. Asian Americans (2%) and Native Americans (1%) constituted very small proportions of the sample. A slight majority of the sample was male (52%); people as young as 20 and as old as 82 were interviewed, and the mean age of interviewees was approximately 50 years.

The informants were interviewed face to face, either in their homes or in public places (e.g., office at a local community organization, informant’s workplace, restaurant). The interviews were tape recorded and generally ranged in length from 1.5 to 2.5 hours.

A semi-structured interview guide was used for the interviews. The full interviews were coded using the QSR NUD*IST program (Qualitative Solutions and Research, Non-numerical Unstructured Data Indexing Searching and Theorizing) and summarized using Filemaker. This study focused on responses to interview questions about the framing of alcohol problems. Respondents were asked to tell the interviewer how they defined alcohol problems and what they viewed as the most important issues or problems related to alcohol in their city or town. A series of codes was developed based on the themes mentioned by the respondents, and more than 60 individual codes emerged through this process. The principal investigator and one other researcher jointly coded all the responses using these categories and came to a consensus about all the codes assigned to each response. This process was used to ensure no errors or differences occurred in the coding based on having different raters. The codes assigned to responses were not mutually exclusive and frequently reflected several kinds of themes. The distribution of responses on the definitions of alcohol problems were compared with those on the importance of alcohol problems using Wilcoxon tests. Regression analyses were used to determine if there were significant differences in themes by activist type or city.

## 3. Results and Discussion

Data from the 60 individual themes that emerged in the initial analysis were grouped into six basic diagnostic framing categories. Several of these categories reflected themes used in major conceptual models; namely, the alcoholism or abusive drinking construct, the individual alcohol-related problems framework and the public health framework for addressing alcohol problems. As predicted, an individual responsibility/criminal sanctions framework did not emerge as a strong theme in this sample. Although respondents in the study recognized individual alcohol-related problems, they tended to view them as a reflection of life problems and/or as the social and physical effects of alcohol and not as evidence of criminality and deviance, as implied in the anti-drunk driver movement. However, many informants described alternate models, which coalesced into three additional themes: social structural problems that were found in inner city neighborhoods and were embedded in or exacerbated by alcohol problems, problems with the normative climate of alcohol use, and alcohol’s role in drug-related problems. Taken together, the six primary diagnostic frameworks used in the following analyses included *individual alcohol-related problems, alcoholism and alcohol abuse, public health approaches to alcohol problems, social structural problems related to alcohol use, problems in normative contexts of alcohol use,* and *drug-related problems*. The frameworks were used as the basis for the three phases of data analysis described in the following sections. They included analyses on the respondents’ definitions of alcohol-related problems, ranking of the relative importance of alcohol problems, and variation in how alcohol problems are ranked or defined according to activist role or community setting. Taken together, these results describe the diagnostic framing characterized by respondents in this study.

### 3.1. Defining Alcohol-Related Problems

#### Individual alcohol-related problems

As shown in Table 1, respondents in the study provided strong support for the disaggregated alcohol-related problems framework, or the notion that drinkers can experience a wide range of problems related to alcohol use that do not stem from alcohol addiction [13]. When defining alcohol problems, three-quarters of respondents described a range of negative consequences experienced by drinkers or their families. Almost half of the respondents (44%) described overall adverse effects of alcohol use on individual drinkers. For example, one respondent from Detroit said alcohol use becomes problematic when it “affects your ability to live life productively, to carry out your responsibilities. . . your ability to function properly, to be able to further your life and your well-being”. In addition, some of the responses described specific problems related to drinking, such as health problems (14%), problems in the workplace or with maintaining a job (13%), and driving under the influence (9%). For example, a respondent from Baltimore said that “on a personal level, if a person has a problem with alcohol. . . it could be a bad history of driving, getting into trouble. . . getting along with other folks because they’re not in their right senses. . . . [Alcohol] causes problems in the family, at the workplace. . . . Any time they drink too much, that causes these problems, then that’s bad”. Another informant from Los Angeles stated, “My personal definition of alcoholism is any kind of problem. You drinkin’ and it is related to your drinking, then you’ve got an alcohol problem. . . A parking ticket because you was drunk and forgot you parked there, whether there was an automobile accident or whether you ran into a building and hurt people and property, which constitutes felony while drinking. . . . Now, either your liver, you got bad nerves, ulcers—all the health issues that come with it are so obvious”. Some of the responses indicated that alcohol is a problem when used by drinkers for self-medication as a way to escape reality and ease psychological pain (13%), or as a way of dealing with depression or low morale (7%). For example, one informant from Detroit defined alcohol problems as hopelessness: “Just no future, no vision, no self-esteem”. She explained that “self-esteem is the way we feel about ourselves; if we feel hopeless, we need an escape or we think we need an escape. [Instead of jogging or working out] we go get a 40-ounce”. Finally, a few respondents defined alcohol problems in relation to being idle or standing around with nothing else to do.

Family and youth alcohol-related problems also were a major concern for respondents in the study. When defining alcohol problems, more than a quarter referred to the harmful effects of alcohol use on family life. The major problems included marital discord and breakups; diverting money from the needs of families and children (e.g., food, shoes, and rent) to support drinking habits; and child neglect and abuse and domestic violence. Echoing some of these themes, a respondent from Oakland stated, “In terms of the community, I see that there is a great deal of domestic violence, and so there are many divorces and many families are abandoned. And I see [as] the result of that, there is very little participation in the children’s schooling. And since there’s not enough money at home, the children often have to look for work instead of going to school. And then their mothers also have to find one or two jobs because welfare doesn’t give enough money. And so I see that alcohol causes all of the disrepair and destruction of the family”. Another respondent from this city talked about the cause-and-effect relationship of alcohol as it relates to husbands who are abusive drinkers. He focused on situations in which a man might “drink up his check”, letting his kids starve and rent go unpaid, which would the cause his spouse to get upset, and be possibly beaten by him.

About 10% of the informants mentioned youth drinking issues when defining alcohol-related problems. Most of the comments were references to underage drinking, problems stemming from youth drinking, or youth drinking environments. The kinds of common problems associated with youth drinking included violence or disruptions at parties and accidents related to driving under the influence. A respondent from Baltimore said, “I know we have a problem in terms of use of alcohol by minors. The statistics in that report [Governor’s Blue Ribbon Commission] are pretty startling about use of alcohol by minors; the dangers associated with that use related to drunk driving, drinking, and boating accidents; violence associated with the use of alcohol, including sexual violence”. One informant from Detroit found that alcohol use is a serious problem among African Americans, particularly young people. He described the regular practice of high school students taking their lunch breaks to buy 40-ounce bottles of alcohol, which they consume in parking lots while socializing with friends, with the full awareness of teachers. The drinking continues with students purchasing alcohol on the way home from school and consuming to the point where drinking on one’s porch and leaving the malt liquor bottles behind “becomes a part of [the] culture of the neighborhoods.

#### Alcoholism and alcohol abuse

Models of alcohol problems based on addictive or abusive drinking were widely discussed by informants in the study. More than half (51%) of respondents defined alcohol problems in these terms. Responses describing alcoholism or addiction (30%) emphasized symptoms such as dependence, loss of control, and tolerance. For example, one informant from Detroit stated that “alcoholics. . . are physically and psychologically dependent on alcohol” and defined alcohol as a drug because users build up a tolerance for it and require increasing amounts before they exhibit signs of intoxication. In Los Angeles, an informant defined alcohol problems as “somebody who is addicted or their life is controlled in a daily acquisition of alcoholic beverages”. An informant from Milwaukee defined alcohol problems in terms of his own experience as an alcoholic, whereby alcohol takes over a person’s life to the point he or she cannot “do anything without alcohol”. A respondent from San Antonio commented on the medical consequences of continual alcohol use and difficulties with withdrawal: “The medical problems that occur from them constantly being exposed to the alcohol is long range. . . . When this person is a alcoholic, it’s hard on detoxification, as opposed to a person who is dependent on a drug like marijuana or dependent on a drug like heroin. The withdrawal symptoms and all the things that person has to go through medically, it could tear you apart. . . . It’s very traumatic when you see somebody that you know going through the withdrawal symptoms of alcohol”.

Aside from addiction, some informants (30%) defined alcohol problems in terms of abusive or excessive drinking. Most often informants described intoxication or drinking to the point that it leads to personality changes or social disruption. An informant from Detroit defined alcohol problems as “when there’s a overindulgence which causes the person who’s consuming the alcohol behavior or mood to change. . . [which] could possibly lead to a violent individual or individual with no rationale or whatever”. A respondent from Los Angeles made a similar point: “Alcohol problems is when a person drinks on a continual basis. . . they must have that 6-pack every night or several cocktails. It can also be a problem for even just a weekend drinker. And, it just changes up the person’s whole personality”. In Milwaukee, a respondent defined alcohol problems as “any use above moderation” that impairs judgment or physical actions, or a negative change in behavior because of the abuse of alcohol.

#### Public health approaches to alcohol problems

A public-health-oriented model of alcohol problems also was widely reflected in responses by informants in the study. Nearly half of those interviewed (46%) discussed one or more issues related to alcohol sales and marketing. Alcohol problems were defined in terms of the overconcentration of alcohol outlets (16%); problems with selling practices and the environment in and around outlets (10%); and challenges with zoning, licensing, and regulating stores (6%). A respondent from Milwaukee focused on the problems of having a high density of alcohol outlets and the problematic selling practices of owners. He mentioned a neighborhood population composed of 63% youth, with almost 30 liquor stores in a 14-block area. He said many business owners knew welfare had been cut in Wisconsin, so they would not take a 6-pack apart and sell single beers at the beginning of the month; however, in the second through fourth weeks, “they know people are going through real hard times, they’ll set up deals and stuff. So they could still make their money and pull you in. . . . And they most definitely try to work with the minors in the neighborhood”. A community leader from Oakland also discussed the problems with outlets, which she said were intensified by the fact that her neighborhood had “five liquor stores within two blocks”. This respondent viewed “alcohol problems as a liquor outlet [includes stores where at least 30 to 40% of sales are in alcohol] and it can be people in bars”. She stated, “I have noticed that whether it’s a momma-and-poppa store on a corner, if they sell alcohol. . . people will go in and buy beverages and come out. And then they’ll stand there and [go] back and forth just getting alcoholic beverages. . . . And then when they get too intoxicated to go about their business. . . they would just either sit down there in front of the store and go to sleep or they stand there and they beg. . . . The fact there they were out there begging was. . . one thing that was bad. . . “cause most of the people in this community are working families or either seniors”. The respondent also mentioned that the families and seniors had to pass youth selling drugs out in front these establishments, litter from the numerous containers of alcohol, and/or people lying or sitting down in the street. A respondent from Los Angeles linked overconcentration with zoning and licensing issues: “Alcohol became a problem when the city planners allowed them to over-saturate it with liquor stores, when the land usage laws were ignored. The city administrators were aware that there was an overconcentration of alcohol. . . but they looked the other way. They haven’t sold any liquor license since 1965; however, liquor stores are popping up all over because the liquor licenses that were sold never expired”.

Easy accessibility to alcoholic beverages through high levels of physical availability (12%), attractive pricing (5%), and aggressive sales and promotion (3%) were other important issues voiced by informants in their definitions of alcohol problems. Respondents linked high levels of alcohol availability to heavy alcohol consumption and to a variety of related problems. Echoing these concerns, one informant from Raleigh said, “The major thing is the sheer amount of alcohol that’s sold in our neighborhood is a problem . . . . The fact that more alcohol is sold than any other legal product. . . that’s a real problem because it encourages alcoholism, domestic abuse, trash, filthiness around the neighborhood”. A respondent from Los Angeles said, “Alcohol [is] usually the most available in communities that can least afford it. And when I say the most available, I mean. . . it’s not unusual on some of the main thoroughfares to find 4 or 5 liquor stores within six blocks”. In Detroit, the director of a community-based organization focused on cheap pricing and convenient packaging of alcohol, which “causes the most devastation”. He stated, “It’s the drug that’s legal, it’s the drug that’s most readily available, and the packaging is such that anybody can afford it. There was a time when those little bitty airline bottles of liquor or booze. . . there was a time when the only place you could buy that was a souvenir shop on an airplane. . . . But now they sell it in the stores all day every day, 75 cents, 50 cents. . . . They went from the fifth. . . [to] the pint. Then they went to the pocket pint, which is smaller than a pint, just large enough to slip right into the ol’ back pocket, whip it out whenever you want, to the airline size. In other words, we’re covering all areas of the market. If you got 50 cents, you could walk in here and get a little swigger to shake the hanks off of you”. In addition to expressing concerns about the high quantities and low prices of beverages available in these communities, some informants focused on characteristics of beverages (alcohol beverage type 7%) they believed to be particularly harmful, such as fortified wines, malt liquor, and 40-ounce portions.

Alcohol advertising and media images (12%) and alcohol-related billboards (5%) also were defined as alcohol problems within these communities. Informants believed the glamorization of alcohol enticed people, especially vulnerable populations, such as youth and the poor, to drink either to achieve status or popularity or to escape from problems. Advertisements from television and billboards were identified as the major vehicles through which these messages were conveyed, and informants believed their influence was enhanced by subliminal seduction. One informant from Baltimore said, “In this community, we have a lotta kids that are teenagers that are doing the 40-thing. It’s so popular because they got the commercials and they used to have the billboards. . . . And these teenagers can’t wait for somebody to buy them wine coolers or beer or whatever it is, whatever the drink of choice is, like Alize. . . . A few people used to drink it. . . but now because the advertisement has pushed it, people are drinking that more often. . . . The advertisement is what pushed these things. . . and it is so obvious, but nobody realizes it”. A respondent from San Antonio remarked that alcohol billboards were very prevalent in low-income areas and kept alcohol fresh in a user’s mind. He described a big sign that stood over a church and showed a fifth of liquor being poured into a glass over ice. To get the sign removed, activists worked for many months and met with local and state politicians. However, after being removed, it was replaced with another alcohol ad “with a pretty lady holding the bottle”. This respondent was particularly concerned about the message to children who saw billboards in a supposedly safe environment. A respondent from Milwaukee criticized the heavy promotion of alcohol on billboards throughout the community and argued that “subliminal seduction” helps entice consumers to purchase alcoholic beverages. In Oakland, one informant cited as an aspect of alcohol problems “a subliminal level that comes through advertisements or romanticism that comes with the illusions of the freedom that alcohol brings”.

Informants in the study were particularly concerned about alcohol promotion activities targeted at vulnerable populations, such as ethnic minorities, low-income groups, or youths (7%). Leaders believed targeting poor or other disadvantaged neighborhoods with excessive advertising and alcohol sale venues, while excluding more powerful and affluent communities from these tactics, was inherently unjust and led to higher rates of alcohol problems and worse social conditions in poorer areas. One respondent from Oakland said, “We’re not upper class. We’re semi lower income. That the alcohol industry targets us big time. They make it quite easy and available to buy your one can of poison. . . . You know that strong shit that they’re out of their mind with one bottle of it, you know. That malt liquor stuff. . . . What an easy target is someone that you know maybe not got a lot of education, that may not have a job, and they set them up for addiction. . . . And they’re starting younger and younger and younger and younger as the years go by”.

#### Social structural problems related to alcohol use

Although few contemporary studies have focused on community and neighborhood problems related to alcohol use, respondents in this study were keenly aware of these problems. More than two-thirds discussed alcohol problems in terms of the impact of alcohol use on broader community life. Within this category, the largest proportion of respondents (28%) suggested alcohol use or related issues (e.g., sales and marketing) were problematic because they had negative effects on the community as a whole and/or could be considered a social problem. For example, one of the activists in Los Angeles stated, “We look at alcohol problems like any other social problem. It has social groups, and we would say that there is a number of different social conditions that create the substance abuse problem; specifically, the alcohol problem in the communities that we work in, which are mostly Latino. So we don’t look at it as an individual issue. We see a tremendous social problem in that it involves thousands and tens of thousands of people, and I’m not just talking about people that abuse, but the people that are victims of domestic violence or economic [problems]”. A number of the informants also defined alcohol problems in terms of the nuisances (e.g., loitering, litter, harassment, and noise; 25%) and anti-social behaviors (e.g., belligerence; 10%) associated with public drinking and its effects on community life. Many of these themes were stated by a respondent from Milwaukee who said, “I define an alcohol problem with people who are either walking up and down the streets, in clearly intoxicated stages; carrying bottles of alcohol. . . whistling or being loud and rude to people; urinating; defecating in public, which happens around here; leaving their bottles; being passed out on your steps. Some drunk drivings you’ll see up and down the street here. Accidents, people vomiting, that’s the outward appearances of an alcohol problem here in this immediate neighborhood”.

A number of activists (14%) defined alcohol problems in terms of the impact of alcohol use on crime in their communities. The most common crimes attributed to alcohol were robbery or stealing; crimes associated with lack of inhibition, such as bar fights and violent confrontations; and prostitution. One informant, a police officer from Los Angeles, described some of these issues: “Ninety percent of the females we arrested were involved with alcohol at the time of arrest, where they’re either inebriated or involved with consuming. . . . And then the people. . . trying to make dates with these girls—most of ‘em are under the influence, so it lowered their inhibitions to go after things like that. . . . Most of the fights that occurred at the bars was abuse of alcohol. People just lose their tempers and [are] out of control and then. . . we have an apparent homicide. All related to this alcohol situation”. An informant from Raleigh focused on neighborhood theft as an issue: “I define it when I see people that are intoxicated hanging around on the streets, not working. At the day care, people break in cars the minute they park ‘em out there. . . . We’ve had several break-ins in the mornings when parents drop children off. I consider that a problem because they have to support their habits and they are not working, so they go out to do whatever they need to do. We’ve had several break-ins in the day care, too”.

Some leaders interviewed (10%) expressed concerns about the impact of alcohol use on local community economies, especially those related to depressed housing values and the lack of economic development in poorer communities. For example, one interviewee from Raleigh stated he was focused on the family “trying to climb out of poverty” who lives on the same block and sees their property value “go to hell” and “who have a 8- or 10-year-old kid that they don’t think they can let play in his own neighborhood”. A respondent from Los Angeles suggested that alcohol consumption, sales, and distribution create environmental problems, such as the focus on alcohol outlets to the exclusion of other types of businesses: “I think a lot of that [lack of commercial development] has to do with people saying, ‘Oh, there’s a lot of liquor stores in the two-block radius. I’m not putting my Starbuck[s] or my Borders Books [there].’” From a related perspective, some informants (8%) described how questionable alcohol marketing and sales tactics or problematic drinking styles were concentrated in poorer or ethnic minority communities, as compared with those in middle-class or suburban environments. Other structural problems (e.g., the presence of blight, racism, educational problems, problems with housing, and lack of alcohol treatment or prevention services) were mentioned, but with less frequency. A somewhat higher percentage of respondents (6%) voiced concerns about the exposure of youth to adverse social conditions in communities as a result of alcohol use or sales and marketing.

#### Problems in normative contexts of alcohol use

Another major issue raised by respondents defining alcohol-related problems was the interrelationship between alcohol use and the social environment (23%). Some leaders interviewed believed permissive social norms (8%), the tendency to ignore or rationalize problems associated with drinking (7%), and the belief that drinking is necessary for recreation or fun (3%) contribute to drinking problems. One respondent from Oakland said, “I can’t speak for other cultures. . . but I can tell you about Latino culture. If you’re going to have a party for a child, if you want everyone to have a good time, the children, the adults. . . many times you would include liquor. You would bring alcohol to the party, and for me, that––well, some years back––that would be very normal. . . . I would see that there would be a birthday or a baptism, and you’d have the piñata for the child, but there would always be liquor. I think that is something that we have to educate our community about. . . because I think that [at] a party for children, there doesn’t necessarily have to [be] liquor there. Although the children won’t drink the alcohol. . . from the time they see this, they grow up seeing it as something normal”. An informant from San Antonio said he does not define alcohol problems from the standpoint of being an alcoholic, but in terms of people overdrinking for recreational purposes: “I just think people use alcohol to an excess. . . . It’s just that people think that alcohol is a way to have fun. And that’s where the misconception is, and the misuse starts”. In addition to these issues, a few respondents defined alcohol problems in terms of the disrespectful behavior of drinkers (2%), as a reflection of spiritual problems (2%), and as a potential danger to the broader social fabric (1%).

#### Drug-related problems

In defining alcohol problems, some informants focused on drug-related issues (17%). Some viewed alcohol use as particularly harmful because it leads to illicit drug use and abuse. For example, an informant from Detroit, a police officer, defined alcohol as an abused drug that leads to the abuse of other drugs: “Alcohol is like the gateway. It’s the formation, the start of, the beginning [of] problems into other substance abuse”. He described the progression from 40-ounces of alcohol to cigarettes and cigars, then on to marijuana, and eventually to heroin and cocaine. This abuse leads to the loss of jobs due to inebriation, inability to function, or failure to pass drug screening tests. Other responses focused on the fact that alcohol is a legal drug (9%) that can be as dangerous as or more easily abused than illegal drugs. These responses often described the joint use of drugs and alcohol or the physical proximity of drug dealing to alcohol outlets. An informant from Baltimore stated, “All of our open-end drug markets are located next to alcohol outlets. . . . Alcohol is the single most abused drug in our neighborhood. It is abused by those persons who are manically depressed. People who are self-medicating [with] alcohol who also use other substances. Crack users kick their high, so to speak, by using malt liquor and malt liquor that is 5 to 6 times higher in potency than the average malt liquor. . . . The illegal drugs are not as abused as alcohol”.

#### 3.2. Ranking Alcohol-Related Problems

After defining alcohol-related problems, informants were asked to describe which alcohol problems they viewed as most important in their community. The respondents mentioned many of the themes described in the previous section; however, substantial and significant differences were found with respect to the prevalence of themes ranked as the most important problems relative to the themes used to define alcohol problems (Table 2). Probably the most dramatic difference was between the percentage of respondents defining alcohol problems in terms of alcoholism (31%), compared with the percentage viewing it as one of the most important problems in their area (7%). A large gap also was found between the percentage defining alcohol problems in terms of abusive or excessive drinking (30%), compared with the percentage stating it was a very important problem (10%). In sum, although half of the leaders defined alcohol problems in terms of alcoholism or abusive drinking, only 17% ranked these problems as among the most important problems in their communities.

A substantial divide also was found between the percentage of informants defining alcohol problems in terms of negative drinking consequences for individuals or families (44%) and the percentage ranking these issues as the most important problems (15%). Moreover, significantly more informants defined alcohol problems as self-medication (14%) and family problems (27%) than saw those as among the most important problems (4% and 15%, respectively). In addition, fewer respondents ranked health problems, job problems, low morale, and idleness as the most important problems than included these factors as part of their definition of alcohol problems, although these differences were not statistically significant. However, informants were as or more likely to regard youth alcohol-related problems (*p* < 0.10) and domestic violence as among the most important alcohol problems as they were to include these factors in their problem definition.

Although respondents deemphasized alcoholism, abusive drinking, and drinking problems in their rankings of important problems, they were just as likely or more likely to focus on themes related to alcohol sales and marketing, community problems, socio-cultural problems and drug-related issues as they were to include these factors when defining alcohol problems. In fact, significantly more respondents stated that youth availability, crime, drug problems, and lack of alcohol education were among the most important problems than discussed these issues when defining alcohol problems. For example, the proportion of respondents ranking crime as among the most important alcohol problems was almost twice as high as the proportion defining alcohol problems in those terms (25% versus 14%), and the proportion regarding drugs as an important problem was 17%, compared with 10% who included drugs as a factor in defining alcohol problems. Respondents were four times more likely to view lack of alcohol education as important rather than to define alcohol problems in those terms (9% versus 2%). A similar trend was found with respect to alcohol outlet regulation and youth concerns. The only major negative discrepancy between defining versus ranking alcohol problems occurred with regard to respondents mentioning general community problems (28% versus 12%). However, this should be considered in conjunction with the finding that a higher percentage of respondents ranked crime and lack of alcohol education among the most important issues than mentioned them in defining alcohol problems.

### 3.3. Variation in Defining and Ranking Alcohol-Related Problems

The analyses explored whether significant differences existed between how alcohol problems were defined and how they were ranked by respondents in different communities and by informants who played different types of roles in community leadership. Because differences existed in the distribution of types of activists across different cities (Table 3), regression analyses were conducted to analyze the joint effect of community site and activist type on each of the problem indices (Tables 4 and 5). The results showed that although relatively few differences were found with respect to how respondents in different cities or roles framed alcohol-related problems, these differences were more likely to occur when defining, rather than ranking, problems. First, informants from Detroit (*p* = 0.016) were significantly more likely than were those from other cities to define alcohol problems in terms of alcohol addiction and abuse, while respondents from Oakland (*p* = 0.021), Raleigh (*p* = 0.007) and Baltimore (*p* = 0.023) were significantly more likely than were those from other cities to define these problems in terms of community issues. In addition, respondents residing in Detroit (*p* = 0.010), San Antonio (*p* = 0.003) and Baltimore (*p* = 0.008) were more likely than were others to mention socio-cultural issues when defining alcohol-related problems. Religious leaders were significantly more likely to define alcohol problems in terms of alcohol sales and marketing (*p* = 0.021) and drug problems (*p* = 0.000) than were other informants.

In terms of ranking alcohol-related problems, politicians (*p* = 0.013) and informants living in Baltimore (*p* = 0.001) were more likely than were others to describe alcohol abuse and alcohol addiction as important alcohol problems, and professional activists (*p* = 0.32) and residents of Raleigh (*p* = 0.0000) were more likely than were others to view community-level problems in this manner.

Although few overall differences were found with respect to how respondents from different areas or in different roles framed alcohol-related problems, the findings suggest that framing processes may be nuanced according to different community contexts or policy goals. For example, respondents in both of the cities that emphasized community-level problems were engaged in policy changes regarding alcohol outlets, and respondents in communities that emphasized the socio-cultural context of alcohol abuse and addiction were engaged in fighting media images of alcohol.

## 4. Conclusions

The findings from this study illustrate that activists engaged in efforts to change alcohol policies in local communities expressed some of the same conceptualizations as did those characterizing the dominant models for framing alcohol problems. Their definitions of alcohol problems reflected some of the assumptions associated with the alcoholism as a disease model, the alcohol problems perspective, and the public health model of alcohol problems. The only model that had little support was the individual responsibility and blame model, described in the drunk driver reform movement. However, the findings also showed the emergence of other significant frameworks not described in the literature. These models focused on the impact of alcohol use on the broader community and neighborhood context, on problems in the normative context of alcohol use, and on problems linking alcohol use with illicit drug use.

When the respondents were asked to rank problems in terms of their importance, the community model of alcohol problems and public health model emerged as the most dominant, followed by the alcohol problems perspective, normative problems and drug problems related to alcohol, and finally models based on alcoholism as a disease or abusive drinking.

As described previously, the goal of the study was to describe the frameworks used by leaders engaged in campaigns to change alcohol policies in inner city areas, and to examine their diagnostic framing in order to better understand how social movements form. Social movement theorists have argued that the framing process mirrors the goals and strategies of activists seeking social change [5,11]. Given that most of the leaders interviewed were engaged in alcohol policy work focused on regulating the sales, marketing, and advertising of alcoholic beverages, it was not surprising that they ranked public health definitions of alcohol problems very highly. However, the strong focus on community problems in both defining and ranking alcohol problems was not anticipated. This framework has not received much attention in the literature, with the notable exception of recent ecological studies linking alcohol outlets with crime, violence, and sexually transmitted diseases [15,17,18]. The dominant frameworks for alcohol problems prevention, like those for other health promotion efforts, have focused on changing individual lifestyles [19]. Although the public health model of alcohol problems has to some extent embraced problems related to the sale and marketing of alcoholic beverages, it has largely ignored the economic and social conditions in inner city neighborhoods in which alcohol is promoted and sold.

In fact, the adverse impact of alcohol at the community and neighborhood level appears to be one of the most salient ways of framing alcohol problems for citizen groups interested in social activism in inner city communities. Based on interviews in this study, the public use of alcohol was perceived to have very negative consequences in areas with high levels of crime, poverty, blight, drug use, and other social problems. A previous analysis from this study [20] showed that leaders became involved in alcohol policy campaigns in part to address long-standing neighborhood problems of crime, disorder, and drug use, which they saw as linked to public drinking as well as to economic underdevelopment. Frustration with these adverse living conditions and the desire for neighborhood improvement prompted local communities to organize around alcohol problems.

The findings from the present study suggest that expanded frameworks of alcohol problems that encompass neighborhood- and community-level effects might be most useful for developing prevention approaches that relate to the experiences of people living in disadvantaged neighborhoods. As described in recent research on alcohol policy [21, 22], the study results also affirm the need for adopting broader public health alcohol policies that include a focus on access to health education and on changing drinking norms, especially for youth. In addition, the findings suggest a need for additional research examining the interrelationships of alcohol availability and other social problems with community economic, political, and social development, such as discussed in the recent work by Theall *et al.* [17] on alcohol availability, infectious disease, and social capital.

## Figures and Tables

**Table 1.** Definitions of alcohol problems.

Definition	Percentage of respondents *N* = 181
**Alcoholism and Alcohol Abuse**	**51%**
Alcoholism, addiction	31%
Abusive or excessive drinking	30%
**Alcohol-Related Problems**	**75%**
**Individual**	
General drinking problems	44%
Health problems	14%
Job problems	13%
Self-medication	13%
DUI	9%
Low morale	7%
Idleness	3%
**Family and Youth**	
Family alcohol problems	27%
Youth alcohol problems	12%
Domestic violence	7%
**Alcohol Sales and Marketing**	**46%**
Overconcentration of alcohol outlets	16%
Alcohol availability	12%
Alcohol advertising and media images	12%
Problems with outlets	10%
Alcohol beverage type	7%
Targeting practices	7%
Alcohol billboards	5%
Alcohol pricing	5%
Alcohol outlet zoning	3%
Sales to minors	3%
Profit motive	3%
Alcohol sales	3%
Youth availability	2%
Alcohol outlet regulations	2%
Alcohol industry	2%
Alcohol licenses	1%
Alcohol sponsorship	1%
**Community Problems**	**70%**
General community problems	28%
Alcohol-related nuisances	25%
Crime	14%
Belligerence	11%
Public drinking	10%
Economic problems	9%
Community comparison	8%
Youth concerns	6%
Blight	3%
Racism	3%
Educational problems	3%
Housing	2%
Safety issues	2%
Government problems	2%
Environmental problems	2%
Lack of alcohol education	2%
Lack of services	2%
Lack of police	1%
Lack of alcohol treatment	1%
Fear	1%
Neglect	0
Unemployment	0
**Socio-Cultural Problems**	**23%**
Norms	8%
Rationalizing	7%
Youth norms	5%
No empowerment	2%
Drinking for fun	3%
Lack of respect	2%
Spiritual problems	2%
Lack of empowerment	1%
Social network problems	1%
**Drug-Related Issues**	**17%**
Drug problems	10%
Alcohol as a legal drug	9%

**Table 2.** Comparison of definitions and rankings for alcohol problems.

	Definition of alcohol problems *N* = 181	Most important alcohol problem *N* = 161
**Alcoholism and Alcohol Abuse**	**51%**	17%***
Alcoholism, addiction	31%	7%***
Abusive or excessive drinking	30%	10%***
**Alcohol-Related Problems**	**75%**	42%**
**Individual**		
General drinking problems	44%	15%
Health problems	14%	9%
Job problems	13%	8%
Self-medication	13%	4%**
DUI	9%	9%
Low morale	7%	6%
Idleness	3%	1%
**Family and Youth**		
Family alcohol problems	27%	15%*
Youth alcohol problems	12%	17%+
Domestic violence	7%	10%
**Alcohol Sales and Marketing**	**46%**	**55%**
Overconcentration of alcohol outlets	16%	19%
Alcohol availability	12%	17%
Alcohol advertising and media images	12%	12%
Problems with outlets	10%	9%
Alcohol beverage type	7%	6%
Targeting practices	7%	9%
Alcohol billboards	5%	4%
Alcohol pricing	5%	6%
Alcohol outlet zoning	3%	2%
Sales to minors	3%	6%
Profit motive	3%	2%
Alcohol sales	3%	3%
Youth availability	2%	7%
Alcohol outlet regulations	2%	4%
Alcohol industry	2%	3%
Alcohol licenses	1%	2%
Alcohol sponsorship	1%	2%
**Community Problems**	**70%**	**64%**
General community problems	28%	12%***
Alcohol-related nuisances	25%	18%
Crime	14%	25%**
Belligerence	11%	7%
Public drinking	10%	10%
Economic problems	9%	12%
Community comparison	8%	6%
Youth concerns	6%	9%+
Blight	3%	6%
Racism	3%	3%
Educational problems	3%	2%
Housing	2%	3%
Safety issues	2%	3%
Government problems	2%	3%
Environmental problems	2%	2%
Lack of alcohol education	2%	9%*
Lack of services	2%	2%
Lack of police	1%	3%
Lack of alcohol treatment	1%	3%
Fear	1%	2%
Neglect	0	2%
Unemployment	0	6%
**Socio-Cultural Problems**	**23%**	**20%**
Norms	8%	12%
Rationalizing	7%	4%
Youth norms	5%	9%
No empowerment	2%	2%
Drinking for fun	3%	1%
Lack of respect	2%	1%
Spiritual problems	2%	1%
Lack of empowerment	1%	1%
Social network problems	1%	1%
**Drug-Related Issues**	**17%**	**20%**
Drug problems	10%	17%*
Alcohol as a legal drug	9%	5%

*** *p* < 0.001

** *p* < 0.01

* *p* < 0.05

+ *p* < 0.10

**Table 3.** Percent distribution of activist types, by city.

	Oakland *N* = 38	Los Angeles *N* = 41	Detroit *N* = 41	San Antonio *N* = 19	Raleigh *N* = 17	Baltimore *N* = 21	Milwaukee *N* = 28
Community activists	24	42	10	26	71	52	43
Professional activists	60	44	50	42	12	10	50
Politicians	13	5	5	5	18	14	7
Clergy	3	7	10	10	0	10	0
Other	0	2	25	16	0	14	0

Chi square = 5.742, *df* = 24, *p* = 0.000.

**Table 4.** Significant predictors in regression models for definitions of alcohol problems by city and activist type

	Alcoholism & alcohol abuse	Alcohol sales and marketing	Community problems	Socio-cultural problems	Drug problems
Politician	--	--	--	ns	ns
Professional	--	--	--	ns	ns
Clergy	--	0.021	--	ns	0.001
Oakland	--	--	0.021	ns	ns
Los Angeles	--	--	ns	ns	ns
Detroit	0.016	--	ns	0.010	ns
San Antonio	--	--	ns	0.003	ns
Raleigh	--	--	0.007	ns	ns
Baltimore	--	--	0.023	0.008	ns
	Adjusted R^2^ = 0.026 df = 1 F = 5.87	Adjusted R^2^ = 0.026 df = 1 F = 5.472	Adjusted R^2^ = 0.076 df = 6 F = 3.470	Adjusted R^2^ = 0.096 df = 9 F = 2.982	Adjusted R^2^ = 0.078 df = 9 F = 1.936

**Table 5.** Significant predictors in regression models for ranking alcohol problems by city and activist type.

	Alcoholism and alcohol abuse	Community problems
Politicians	0.013	ns
Professionals	ns	0.032
Clergy	ns	ns
Oakland	ns	ns
Los Angeles	ns	ns
Detroit	ns	ns
San Antonio	ns	ns
Raleigh	ns	0.00
Baltimore	0.001	ns
	Adjusted *R^2^* = 0.108 *df* = 9 *F* = 3.008	Adjusted *R^2^* = 0.077 *df* = 9 *F* = 2.381
